# Comparison between traditional and new obesity measurement index for screening metabolic associated fatty liver disease

**DOI:** 10.3389/fendo.2023.1163682

**Published:** 2023-04-21

**Authors:** Hongyan Wang, Yuxue Zhang, Yupeng Liu, Hui Li, Ruiling Xu, Hongmei Fu, Chaoqi Yan, Bo Qu

**Affiliations:** ^1^ Department of International Physical Examination and Health Center, Second Affiliated Hospital of Harbin Medical University, Harbin, China; ^2^ Department of Hygiene Microbiology, School of Public Health, Harbin Medical University, Harbin, China; ^3^ Department of Epidemiology and Health Statistics, Institute for Public Health and Management of Wenzhou Medical University, Wenzhou, China; ^4^ Department of Gastroenterology, Second Affiliated Hospital of Harbin Medical University, Harbin, China

**Keywords:** MAFLD, obesity measurement index, body mass index, lipid accumulation product, screening ability, screening strategy

## Abstract

**Objectives:**

Obesity measurement indexes have certain screening value for metabolic diseases. To investigate associations between metabolic associated fatty liver disease (MAFLD) and obesity measurement indexes, including traditional indexes (BMI, WC, WHtR) and new indexes (ABSI, BRI, VAI, LAP), and assess their screening ability.

**Methods:**

12,658 subjects aged 18-75 at the Health Center of a Class III Grade A Hospital were included, who were divided into MAFLD and non-MAFLD groups. Spearman’s rank correlation was used to study the correlation between MAFLD and obesity measurement indexes. Receiver operating characteristic (ROC) curves were used to calculate the area under the curve (AUC) to evaluate their screening accuracy.

**Results:**

MAFLD had strong correlation with traditional BMI and new index LAP. ROC analysis showed that BMI had the highest AUC (0.89), followed by LAP (0.87). Stratification by BMI, LAP had the highest AUC (0.90) for MAFLD in population without obesity (BMI< 23kg/m^2^), and its optimal cutoff value was 20.75, with a sensitivity and specificity of 85.9% and 79.0%, respectively.

**Conclusions:**

We proposed a two-step screening strategy for MAFLD, combining BMI and LAP, and defined a high-risk population for MAFLD as follows: 1) BMI ≥ 23 kg/m^2^; and 2) BMI< 23 kg/m^2^ and LAP ≥ 20.75.

## 1 Introduction

Nonalcoholic fatty liver disease (NAFLD) is the most common liver disease, with a worldwide prevalence of 25%. NAFLD is estimated to affect approximately 173-310 million (12.5-22.4%) people in China. The prevalence of NAFLD in China has increased from 17% (2003) to 22.4% (2012), which is comparable to that in the US (24.13%), Europe (23.71%) and Japan (25%) according to the latest global burden of liver disease data in 2019. In general, NAFLD has become the primary cause of liver disease burden in many countries and regions (1). NAFLD is a term used to describe metabolic dysfunction associated with liver disease and is closely related to genetic susceptibility, insulin resistance, type 2 diabetes (T2DM), metabolic syndrome (MetS), cardiovascular disease and so on. NAFLD can lead to death from not only liver diseases such as liver cirrhosis and hepatic carcinoma but also cardiovascular disease and extrahepatic malignant carcinoma, which seriously threatens human health and places a very large economic burden on society (2). Given that metabolic dysfunction better represents the heterogeneity of NAFLD. In early 2020, a panel of international experts from many countries and regions and the Asian-Pacific Association for the study of the Liver (APASL) proposed renaming NAFLD to metabolic associated fatty liver disease (MAFLD) and developed new diagnostic criteria for MAFLD. The criteria are based on evidence of hepatic steatosis, in addition to one of the following three criteria, namely, overweight/obesity, presence of T2DM, or evidence of metabolic dysregulation (3–5).

Human morphologic or obesity measurement indexes can reflect the degree of obesity and have certain screening value for metabolic diseases (6–9). In addition to body mass index (BMI), there are many other obesity measurement indexes, including traditional indexes such as waist circumference (WC), waist-hip ratio (WHR) and waist-to-height ratio (WHtR), as well as new indexes that have emerged in recent years, such as a body shape index (ABSI), body roundness index (BRI), visceral fat index (VAI) and lipid accumulation product (LAP) (10–13). In recent years, a small number of studies have explored the correlation between obesity measurement indexes and NAFLD, demonstrating the screening value of the new obesity measurement indexes for NAFLD (14, 15). However, since the concept of MAFLD was proposed in 2020, there have been few studies on the correlation between the obesity measurement index and MAFLD. The screening value of these indexes for MAFLD is unclear and deserves further exploration. Therefore, this study mainly utilized adult health examination big data from the Physical Examination and Health Center of Class III Grade A Hospital in a city in northeast China to explore the correlation between the above obesity measurement indexes and MAFLD and to compare the screening abilities of these indexes for MAFLD.

## 2 Methods

### 2.1 Study design and subjects

This study was a retrospective study. Physical examination data derived from the International Physical Examination and Health Center of a Class III Grade A Hospital (Class III Grade A hospitals represent the highest level of classification in mainland China. They are capable of providing high-level medical and health services, and they also undertake higher education and scientific research tasks within their local region and surrounding areas) in a city in northeast China from January to December 2021 were collected. The inclusion criteria were as follows: 1) adults aged 18-75 years and 2) subjects whose physical examination included general physical examination, laboratory tests and abdominal ultrasound. General physical examination included height, weight, waist circumference (WC), systolic blood pressure (SBP) and diastolic blood pressure (DBP). Laboratory tests included total cholesterol (tCHO), low-density lipoprotein cholesterol (LDL-C), high-density lipoprotein cholesterol (HDL-C), triglycerides (TGs) and fasting plasma glucose (FPG). The exclusion criteria were as follows: 1) patients with a history of liver surgery, liver cirrhosis, liver congestion, liver parasitosis, polycystic liver, portal system diseases, unclear nature of occupying lesions of the liver and other serious diseases of gallbladder, bile duct and pancreas; 2) subjects whose liver ultrasound diagnosis conclusion was not clear; 3) subjects who had incomplete physical examination data or error data; and 4) subjects who could not be accurately diagnosed with MAFLD according to existing physical examination data due to lack of homeostasis model assessment of insulin resistance index (HOMA-IR) and high-sensitivity C-reactive protein (hs-CRP), which were not routinely carried out in the Health Center. According to the inclusion and exclusion criteria listed above, a total of 12,658 subjects were included in this study (**Supplementary Material_Flow Chart**). The subjects were divided into a group with MAFLD and a group without MAFLD. MAFLD was diagnosed according to APASL clinical practice guidelines for the diagnosis and management of MAFLD as described as below (5). Written consent from subjects was waived because their data were retrospectively and anonymously extracted from the electronic information system of the hospital. This study was approved by the ethics committee of the Second Affiliated Hospital of Harbin Medical University (KY2022-058).

### 2.2 MAFLD diagnostic criteria

The diagnosis of MAFLD was based on the detection of liver steatosis by ultrasound together with the presence of at least one of three criteria that includes overweight/obesity, T2DM and clinical evidence of metabolic dysfunction (3–5). Overweight/obesity is defined as BMI ≥23 kg/m^2^ by Asian standards; T2DM is defined according to widely accepted international criteria; evidence of metabolic dysfunction includes the presence of at least two metabolic risk abnormalities: 1) WC ≥90/80 cm in Asian males and females; 2) SBP/DBP ≥130/85 mmHg or specific drug treatment; 3) TGs≥1.7 mmol/L or specific drug treatment; 4) HDL-C<1.0 mmol/L for males and<1.3 mmol/L for females or specific drug treatment; 5) prediabetes (i.e., fasting glucose level 5.6-6.9 mmol/L, or 2-hour postload glucose levels 7.8-11.0 mmol/L or HbA1c 5.6-6.4%; 6) HOMA-IR ≥2.5; 7) hs-CRP >2 mg/L. Since HOMA-IR and hs-CRP were not routinely carried out in the Health Center, 543 subjects could not be accurately diagnosed with MAFLD according to existing physical examination data and were excluded from the analysis.

### 2.3 Physical examination data collection

The physical examination records of adults aged 18-75 years who underwent physical examination in the Health Center of a Class III Grade A Hospital in a city in northeast China from January to December 2021 were reviewed through the electronic information system of the Health Center. Basic information, general physical examination, laboratory tests and abdominal ultrasound data were collected. The basic information included sex and age; the general physical examination included height, weight, WC, SBP and DBP; and the laboratory tests included tCHO, LDL-C, HDL-C, TGs and FPG. Different obesity measurement indexes, including BMI, WHtR, ABSI, BRI, VAI and LAP, were calculated using the following formulas:


(1) $$BMI\left(kg/m^{2}\right)=Weight\left(kg\right)/Height^{2}\left(m^{2}\right)$$

(2) WHtR=WC (cm)/Height (cm)

(3) $$ABSI=WC\;(m)/[BMI\;{\left(kg/m^{2}\right)}^{2/3}\times Height\;{\left(m\right)}^{1/2}]$$

(4) $$BRI=364.2-365.5\times {\left[1-{\left(WC\;\left(m\right)/2\pi \right)}^{2}/{\left(0.5\times Height\;\left(m\right)\right)}^{2}\right]}^{1/2}$$

(5) LAP (male)=[WC (cm)−65]×TG (mmol/L),LAP (female)=[WC (cm)−58]×TG (mmol/L)

(6) $$\begin{array}{l} VAI\;\left(male\right)=\left[WC\;\left(cm\right)/\left[39.68+1.88\times BMI\;\left(kg/m^{2}\right)\right]\right]\\ \times \left[TG\;\left(mmol/L\right)/1.03\right]\times \left[1.31/HDL-C\;(mmol/L)\right],\\ VAI\;\left(female\right)=\left[WC\;\left(cm\right)/\left[36.58+1.89\times \mathrm{BMI}\;\left(kg/m^{2}\right)\right]\right]\\ \times \left[TG\;\left(mmol/L\right)/0.81\right]\times \left[1.52/HDL-C\;(mmol/L)\right] \end{array}$$

### 2.4 General physical examination

Weight and height were measured by an ultrasonic height and weight measuring instrument (SG-1000SC, Beijing Chioy Medical Technology Co., LTD) with the examinee wearing light clothing and no shoes. WC was measured around the abdomen with a soft tape parallel to the floor halfway between the lower rib and the iliac crest when the examinee relaxed and exhaled normally. Blood pressure was measured using an automatic medical electronic sphygmomanometer (ABP-1000, Beijing Chioy Medical Technology Co., LTD) after the examinee rested quietly for at least 5 minutes, and the values of SBP/DBP were recorded.

### 2.5 Laboratory tests

All physical examinees fasted at least 8 hours, and blood samples were collected by the registered nurse in the Health Center. All blood samples were tested by the department of clinical laboratory in the hospital. tCHO, LDL-C, HDL-C, TGs and FPG were detected by an auto biochemical analyzer (Roche MODULAR ISE900, Switzerland).

### 2.6 Ultrasound assessment of fatty liver

All physical examinees fasted at least 8 hours. Ultrasound examination was performed using an ultrasonography instrument (Siemens 2000, Germany) equipped with a curved array transducer probe (4-8 MHz). The ultrasound measurements were conducted by an accredited sonographer. Fatty liver was assessed according to the echogenicity of the liver parenchyma, the visibility of the vascular structure and the clarification of the diaphragm.

### 2.7 Statistical analysis

In this study, we included all individuals who met the inclusion and exclusion criteria during 2021. We used PASS (version 11.0.7) to calculate the statistical power of our analyses. The sample size of 6911 MAFLD subjects and 5747 non-MAFLD subjects in this study achieve 100% statistical power to detect AUCs between 0.70 and 0.90 using a two-sided z-test at the significance level of 0.05.

Statistical analyses were conducted using SPSS (version 19.0). Measurement data were analyzed by Kolmogorov−Smirnov normality tests; data that presented a nonnormal distribution are expressed as the median (lower quartile to upper quartile) [M (P25-P75)], and the Mann−Whitney U test was used for comparisons between groups. Count data are expressed as the frequency (rate) [n (%)], and the χ^2^ test was used for comparisons between groups. After sex stratification, Spearman’s rank correlation was used to assess the correlation between MAFLD and traditional and new obesity measurement indexes separately. Correlation coefficients (r) of 0.8-1.0, 0.6-0.8, 0.4-0.6, 0.2-0.4 and<0.2 were defined as very strong, strong, moderate, weak, and very weak correlation or no correlation, respectively. Receiver operating characteristic (ROC) curves were used to calculate the area under the curve (AUC) and 95% confidence interval (95% CI). The Z test was used to test the statistical significance of the AUCs for each index, and the optimal cutoff value and its corresponding sensitivity and specificity were determined according to Youden’s index. All reported *p* values were two-tailed, and the level of statistical significance was set at 0.05.

## 3 Results

### 3.1 Characteristics of study subjects and prevalence of MAFLD

**Figure 1:**
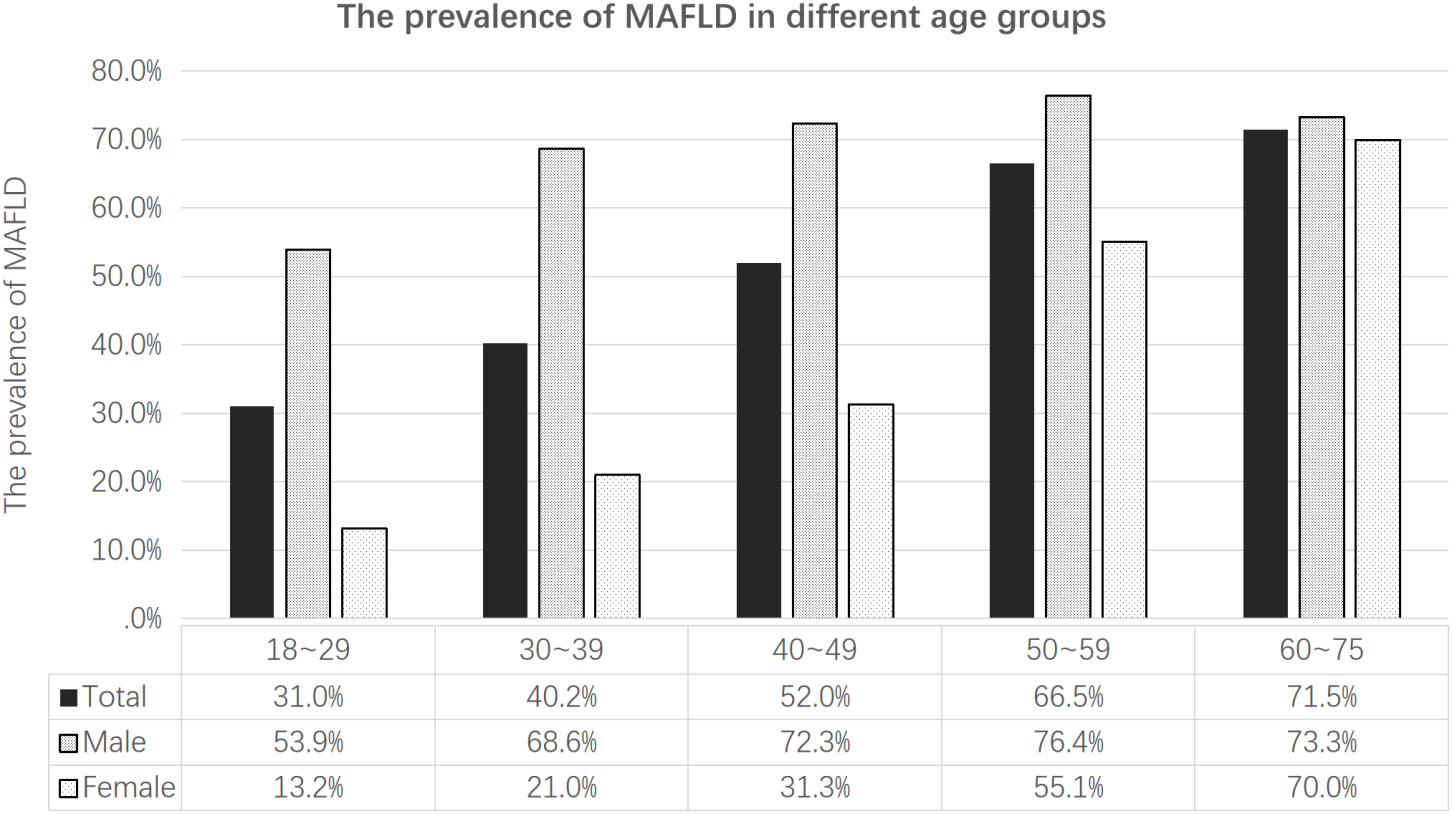
The prevalence of MAFLD in different sex and age groups.

A total of 12,658 subjects, including 5952 males and 6706 females, were included in this study. Overall, the prevalence of MAFLD was 54.48% (6911/12658). The prevalence of MAFLD in males was significantly higher than that in females (71.93% vs. 39.22%) (*p*<0.001). The prevalence of MAFLD in females gradually increased with age, but the prevalence of MAFLD in males was the highest among individuals between 50 and 59 years old. Although the prevalence of MAFLD in males declined slightly after 60 years of age, the overall prevalence of MAFLD was 70% or higher in both males and females after 60 years of age (as shown in **Figure 1**). The clinical characteristics of the subjects are shown in **Table 1**. The clinical values (SBP, DBP, tCHO, LDL-C, TGs and FPG) of the subjects with MAFLD were higher than those in individuals without MAFLD, but HDL-C in the MAFLD group was lower than that in the non-MAFLD group (*p*<0.001). Obesity measurement indexes (WC, WHtR, BMI, BRI, VAI, LAP) in the MAFLD group were higher than those in the non-MAFLD group (*p*<0.001), but ABSI showed no significant difference between the two groups (*p=*0.40).

**Table 1:** Basic characteristics of study subjects.

Variables	Total (n=12658)	non-MAFLD (n=5747)	MAFLD (n=6911)	Z/χ2	p
Demographic data characteristics					
Male (%)	5952 (47.02%)	1671 (28.07%)	4281 (71.93%)	1360.772	<0.001
Female (%)	6706 (52.98%)	4076 (60.78%)	2630 (39.22%)		
Age (years)	46 (37-57)	41 (34-51)	50 (40-59)	-30.26	<0.001
Clinical data characteristics					
SBP (mmHg)	123 (112-137)	116 (107-127)	130 (119-143)	-44.42	<0.001
DBP (mmHg)	77 (69-85)	72 (65-79)	81 (74-89)	-45.27	<0.001
t-CHO (mmol/L)	4.88 (4.29-5.53)	4.70 (4.18-5.32)	5.05 (4.43-5.70)	-18.937	<0.001
LDL-C (mmol/L)	2.97 (2.47-3.52)	2.83 (2.35-3.34)	3.11 (2.58-3.65)	-18.361	<0.001
HDL-C (mmol/L)	1.26 (1.07-1.50)	1.43 (1.22-1.65)	1.15 (1.00-1.33)	-49.016	<0.001
TGs (mmol/L)	1.32 (0.90-1.99)	0.96 (0.72-1.32)	1.74 (1.24-2.44)	-57.835	<0.001
FPG (mmol/L)	5.18 (4.84-5.64)	4.99 (4.72-5.31)	5.37 (5.00-5.97)	-38.93	<0.001
Obesity measurement indexes					
BMI (kg/m2)	24.39 (21.94-26.84)	21.80 (20.09-23.66)	26.28 (24.54-28.40)	-75.363	<0.001
WC (cm)	83 (77-90)	77 (70-83)	87 (83-94)	-65.882	<0.001
WHtR	0.491 (0.459-0.523)	0.458 (0.429-0.486)	0.512 (0.490-0.546)	-67.277	<0.001
ABSI	0.076 (0.072-0.079)	0.075 (0.073-0.079)	0.076 (0.720-0.080)	-0.841	0.40
BRI	4.12 (3.74-4.53)	3.80 (3.49-4.17)	4.36 (4.04-4.77)	-54.564	<0.001
VAI	1.61 (1.00-2.61)	1.10 (0.77-1.63)	2.19 (1.47-3.39)	-56.138	<0.001
LAP	28.65 (15.66-49.59)	15.64 (9.74-25.08)	43.56 (28.92-66.24)	-71.529	<0.001

MAFLD, metabolic associated fatty liver disease; non-MAFLD, without metabolic associated fatty liver disease; SBP, systolic blood pressure; DBP, diastolic blood pressure; tCHO, total cholesterol; LDL-C, low-density lipoprotein cholesterol; HDL-C, high-density lipoprotein cholesterol; TGs, triglycerides; FPG, fasting plasma glucose; BMI, body mass index; WC, waist circumference; WHtR, waist-height-ratio; ABSI, a body shape index; BRI, body roundness index; VAI, visceral adiposity indicators; LAP, lipid accumulation product. Data were expressed in form of Median (upper and lower quartile) [M(P25~P75)] or Frequency (rate) [n (%)]. n refers to the total number of each group.

### 3.2 The prevalence of MAFLD according to quartiles of different obesity measurement indexes

**Figure 2:**
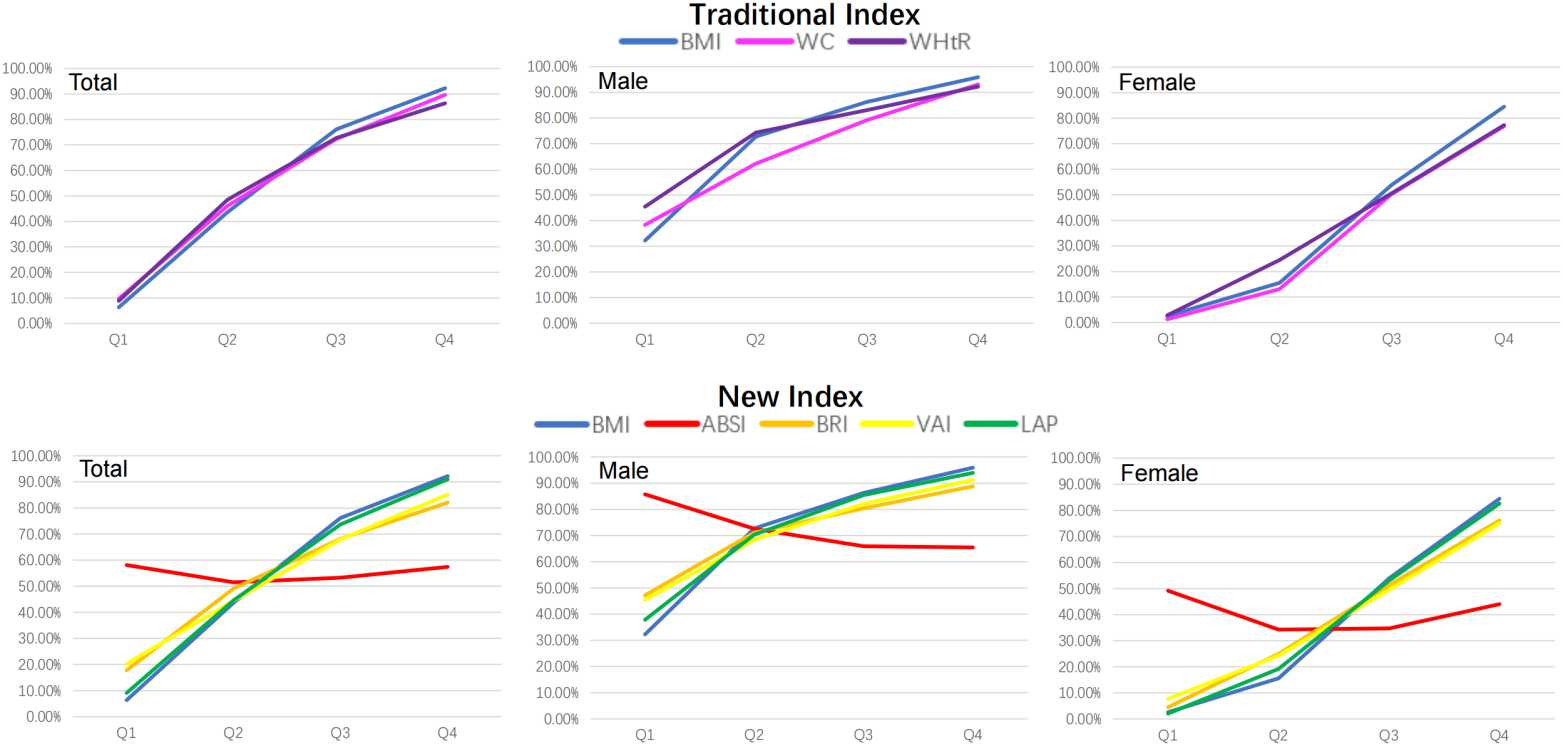
The prevalence trends of MAFLD according to quartiles of seven obesity measurement indexes. BMI was used as a reference standard. BMI, body mass index; WC, waist circumference; WHtR, waist-height-ratio; ABSI, a body shape index; BRI, body roundness index; VAI, visceral adiposity indicators; LAP, lipid accumulation product.

Seven obesity measurement indexes, including BMI, WC, WHtR, ABSI, BRI, VAI and LAP, were grouped according to their quartiles, and the prevalence of MAFLD in the quartiles of these indexes is shown in **Table 2**. The prevalence of MAFLD both in males and females increased linearly with quartile increases in BMI, WC, WHtR, BRI, VAI and LAP. However, the prevalence of MAFLD in males decreased linearly from Q1 to Q3 and leveled off from Q3 to Q4 as the ABSI quartile area increased, and the prevalence of MAFLD in females had no linear increasing or decreasing trend as the ABSI quartile increased (as shown in **Figure 2**).

**Table 2:** Prevalence of MAFLD according to quartiles of seven obesity measurement indexes.

Group	Quartile	Traditional Indexes			New Indexes			
		BMI	WC	WHtR	ABSI	BRI	VAI	LAP
Total	Q1	6.33%	9.68%	8.86%	58.11%	17.77%	20.21%	9.11%
(n=12658)	Q2	43.67%	46.11%	48.51%	51.46%	49.10%	44.45%	44.55%
	Q3	76.16%	72.35%	72.76%	53.23%	68.17%	67.93%	73.71%
	Q4	92.20%	89.61%	86.35%	57.38%	82.05%	85.07%	91.02%
Male	Q1	32.24%	38.31%	45.46%	85.76%	47.17%	45.50%	37.86%
(n=5952)	Q2	72.75%	62.21%	74.34%	72.55%	71.16%	68.36%	70.39%
	Q3	86.31%	79.23%	83.12%	65.92%	80.37%	82.10%	85.45%
	Q4	95.92%	93.07%	92.26%	65.49%	88.82%	91.25%	93.95%
Female	Q1	2.59%	1.22%	2.81%	49.14%	4.49%	7.65%	2.03%
(n=6706)	Q2	15.55%	13.08%	24.45%	34.26%	24.99%	24.26%	19.20%
	Q3	53.95%	50.31%	50.61%	34.69%	51.24%	49.56%	53.07%
	Q4	84.49%	76.93%	77.28%	44.02%	76.13%	75.10%	82.58%

BMI, body mass index; WC, waist circumference; WHtR, waist-height-ratio; ABSI, a body shape index; BRI, body roundness index; VAI, visceral adiposity indicators; LAP, lipid accumulation product. Quartiles, Q1, p0-p25; Q2, p25-p50; Q3, p50-p75; Q4, p75-p100. n refers to the total number of each group.

### 3.3 Correlation analysis between MAFLD and different obesity measurement indexes

Spearman’s rank correlation analysis showed that there was a very negative weak correlation in males (r=-0.17, *p*<0.001) and no correlation in females (*p*>0.05) between ABSI and MAFLD. There was a positive correlation between the other six indexes and MAFLD. Overall, MAFLD had a strong correlation with the traditional index BMI and the new index LAP (r>0.6) and had a moderate correlation with the traditional index WC, WHtR, the new index VAI and BRI (0.4<r<0.6). After stratifying by sex, the correlation coefficients between the other six indexes and MAFLD were as follows, ranked from high to low: BMI (0.54) >LAP (0.48) > WHtR (0.43) >WC (0.42) >VAI (0.39) >BRI (0.36) in males and BMI (0.66) >LAP (0.65) >WHtR (0.60) > WC (0.59) >BRI (0.57) >VAI (0.54) in females. In summary, in males, there was a moderate correlation between BMI, WHtR, WC, LAP and MAFLD (0.4<r<0.6) and a weak correlation between VAI, BRI and MAFLD (r<0.4). In females, there was a strong correlation between BMI and LAP and MAFLD (r>0.6) and a moderate correlation between WHtR, WC, VAI, and BRI and MAFLD (0.4<r<0.6) (**Supplementary Material**).

### 3.4 Comparison of the screening ability of different obesity measurement indexes for MAFLD

**Figure 3:**
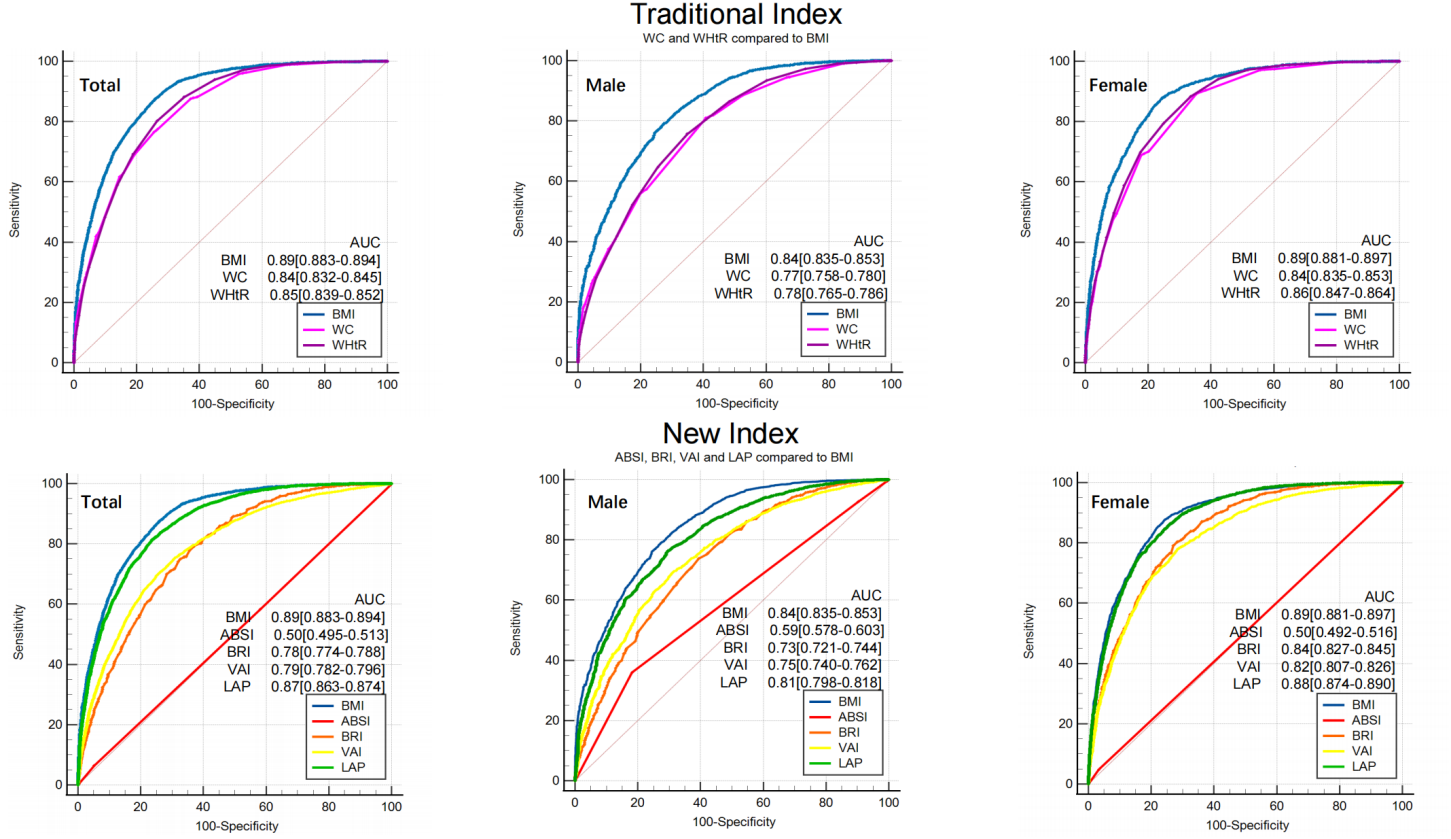
ROC of seven obesity measurement indexes for screening MAFLD. BMI was used as a reference standard. BMI, body mass index; WC, waist circumference; WHtR, waist-height-ratio; ABSI, a body shape index; BRI, body roundness index; VAI, visceral adiposity indicators; LAP, lipid accumulation product. AUC, area under the curve.

ROC curves of seven obesity measurement indexes to distinguish MAFLD were drawn (as shown in **Figure 3**), and their AUCs were calculated (as shown in **Table 3**). After stratification by sex, the AUCs of the seven obesity measurement indexes were ranked from high to low as follows: BMI (0.84) >LAP (0.81) >WHtR (0.78) >WC (0.77) >VAI (0.75) >BRI (0.73) >ABSI (0.59) in males and BMI (0.89) >LAP (0.88) >WHtR (0.86) >WC (0.84) >BRI (0.83) >VAI (0.82) >ABSI (0.50) in females. Among the seven indexes, ABSI had the lowest accuracy for MAFLD (AUC 0.59 for males and 0.50 for females). The AUCs of the other six indexes were all higher than 0.7, which had certain accuracy and certain predictive or screening value for MAFLD. Overall, except for ABSI, the accuracy of the other six indexes for screening MAFLD in females was higher than that in males. The traditional BMI had the best screening ability for MAFLD, with the highest accuracy (AUC 0.84 for males and 0.89 for females), and the optimal cutoff value was 24.74 kg/m^2^ in males and 23.04 kg/m^2^ in females. The new index LAP had better accuracy (AUC 0.81 for males and 0.88 for females), with an optimal cutoff value of 30.85 in males and 20.79 in females. Compared with BMI, the AUC of the other six indexes was statistically significant (*p*<0.001). However, there was no statistically significant difference between LAP and BMI in females (*p=*0.14) (**Supplementary Material**).

**Table 3:** ROC analysis of seven obesity measurement indexes for screening MAFLD.

Group	AUC (95% CI)	Optimal cutoff points	Sensitivity%	Specificity%	Youden’s index
Total (n=12658)					
BMI	0.89 (0.883-0.894)	23.62	86.8	74.8	0.62
WC	0.84 (0.832-0.845)	82	76.6	74.4	0.51
WHtR	0.85 (0.839-0.852)	0.48	80.2	73.5	0.54
ABSI	0.50 (0.495-0.513)	0.08	6.4	94.8	0.01
BRI	0.78 (0.774-0.788)	4.04	74.9	67.5	0.42
VAI	0.79 (0.782-0.796)	1.46	75.4	69.0	0.44
LAP	0.87 (0.863-0.874)	25.75	81.3	76.4	0.58
Male (n=5952)					
BMI	0.84 (0.835-0.853)	24.74	79.0	72.7	0.52
WC	0.77 (0.758-0.780)	86	81.0	59.2	0.40
WHtR	0.78 (0.765-0.786)	0.49	75.7	65.1	0.41
ABSI	0.59 (0.578-0.603)	0.07	36.0	81.9	0.18
BRI	0.73 (0.721-0.744)	3.99	73.8	60.6	0.34
VAI	0.75 (0.740-0.762)	1.59	69.5	68.5	0.38
LAP	0.81 (0.798-0.818)	30.85	76.1	70.6	0.47
Female (n=6706)					
BMI	0.89 (0.881-0.897)	23.04	87.5	75.9	0.63
WC	0.84 (0.835-0.853)	76	89.2	64.7	0.54
WHtR	0.86 (0.847-0.864)	0.46	88.3	66.4	0.55
ABSI	0.50 (0.492-0.516)	0.08	4.9	96.7	0.02
BRI	0.83 (0.827-0.845)	4.11	80.3	71.8	0.52
VAI	0.82 (0.807-0.826)	1.46	77.9	71.6	0.49
LAP	0.88 (0.874-0.890)	20.79	86.1	74.2	0.60

BMI, body mass index; WC, waist circumference; WHtR, waist-height-ratio; ABSI, a body shape index; BRI, body roundness index; VAI, visceral adiposity indicators; LAP, lipid accumulation product. AUC, area under the curve; 95% CI, 95% confidence interval; Youden’s index=Sensitivity+Specificity-1. n refers to the total number of each group.

### 3.5 Comparison of the screening ability of different obesity measurement indexes for MAFLD in populations with or without obesity

**Figure 4:**
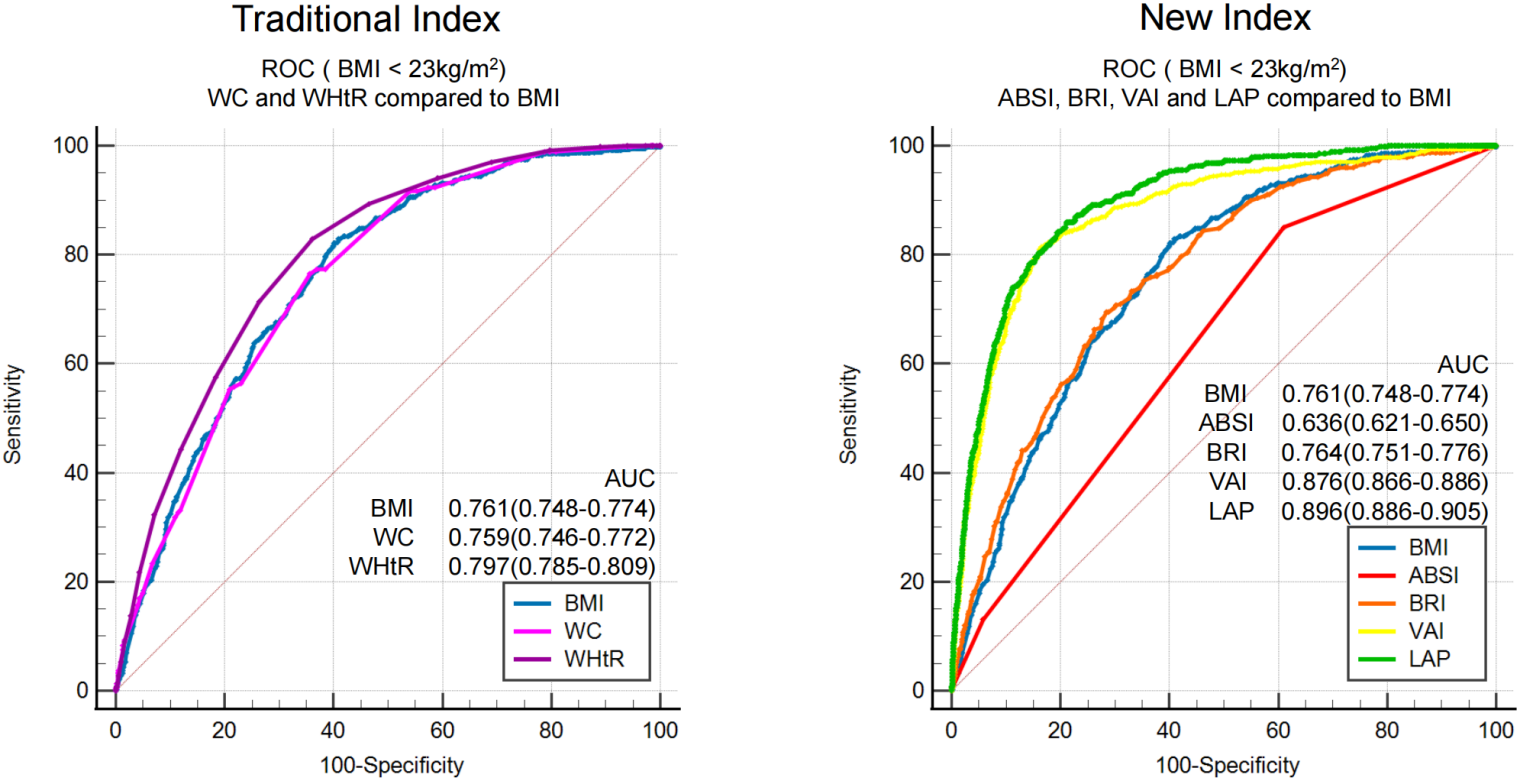
ROC analysis of seven obesity measurement indexes for screening MAFLD in populations with or without obesity.

To compare the screening ability of different obesity measurement indexes for MAFLD in populations with or without obesity, ROC analysis was stratified by BMI. BMI was grouped according to ≥ 23 kg/m^2^ and< 23 kg/m^2^, defined as obesity and non-obesity, respectively, by Asian standards. The proportion of subjects without obesity in this study was 34% (4304/12658). The prevalence of MAFLD in subjects without obesity was 10.87%. ROC curve analysis (**Figure 4**) showed that the AUCs of the seven obesity measurement indexes for MAFLD in subjects without obesity were ranked from high to low as follows: LAP (0.90) >VAI (0.88) >WHtR (0.80) >BRI (0.764) >BMI (0.761) >WC (0.76) >ABSI (0.64) (as shown in **Table 4**). In addition to ABSI, the AUCs of the other six indexes were all higher than 0.7, which had certain accuracy and certain predictive or screening value for MAFLD in populations without obesity. However, the new index LAP had the highest AUC of 0.90 (0.886-0.905); the optimal cutoff value was 20.75, and the sensitivity and specificity were 85.9% and 79.0%, respectively. The AUC of LAP was significantly better than those of the other indexes in predicting MAFLD in populations without obesity (*p*<0.01).

**Table 4:** ROC analysis of seven obesity measurement indexes for screening MAFLD in populations with or without obesity.

Group	AUC (95% CI)	Optimal cutoff points	Sensitivity%	Specificity%	Youden’s index
BMI≥23 (n=8354)					
BMI	0.75 (0.738-0.757)	25.39	95.7	15.4	0.37
WC	0.69 (0.689-0.709)	86	65.0	64.4	0.29
WHtR	0.70 (0.693-0.712)	0.49	71.5	57.7	0.29
ABSI	0.53 (0.514-0.536)	0.07	52.5	51.9	0.04
BRI	0.64 (0.628-0.649)	4.11	71.4	48.3	0.20
VAI	0.72 (0.705-0.725)	1.75	63.6	68.7	0.32
LAP	0.76 (0.753-0.772)	30.75	72.2	66.8	0.39
BMI<23 (n=4304)					
BMI	0.76 (0.748-0.774)	21.13	82.1	59.9	0.42
WC	0.76 (0.746-0.772)	76	76.5	64.3	0.41
WHtR	0.80 (0.785-0.809)	0.45	82.3	64.8	0.47
ABSI	0.64 (0.625-0.654)	0.07	81.2	43.9	0.25
BRI	0.76 (0.751-0.776)	3.90	69.4	71.6	0.41
VAI	0.88 (0.866-0.886)	1.74	81.4	83.6	0.65
LAP	0.90 (0.886-0.904)	20.75	85.9	79.0	0.65

BMI, body mass index; WC, waist circumference; WHtR, waist-height-ratio; ABSI, a body shape index; BRI, body roundness index; VAI, visceral adiposity indicators; LAP, lipid accumulation product; AUC, area under the curve, 95% CI, 95% confidence interval; Youden’s index=Sensitivity+Specificity-1; n refers to the total number of each group.

## 4 Discussion

At present, large-scale epidemiological studies on MAFLD are lacking, but NAFLD can be referred. A recent study based on a comprehensive search of the literature from 1999 to 2018 revealed an alarming national prevalence of NAFLD in China (29.6%). NAFLD prevalence is parallel with urbanization and industrialization. During the past two decades, the burden of NAFLD has increased substantially with the rapid development of economy and radical modifications in lifestyle in China. According to the statistics from the World Bank, the relative increases of national gross domestic product (GDP) per capita were in line with the pooled annual prevalence of NAFLD. This suggested that the epidemic of NAFLD is associated with its economic growth (16). But it’s worth noting that NAFLD or MAFLD is a heterogeneous disease, its epidemiology also relates to sex, age, race, genetic variations, mild to moderate alcohol consumption, obesity, metabolism, lifestyle and educational level (17). With uneven economic development, different regional cultures and diverse lifestyles among the different provinces in China, the epidemiology of NAFLD or MAFLD has shown remarkable regional differences. A recent meta-analysis showed that the incidence of NAFLD is higher in northern China (35.78%) and lower in southeastern China (21.52%). Among provinces in northern China, Heilongjiang has the highest incidence, with up to 50.48% (18). This study was carried out in Harbin of Heilongjiang Province. The prevalence of MAFLD in the healthy physical examination population was 54.48% in this study, which was consistent with previous literature reports. The prevalence of MAFLD in Heilongjiang was significantly higher than the average prevalence in China. On the one hand, northeast China is located in a cold region with a long winter, and a cold climate has a great impact on lifestyle; thus, local residents tend to eat a high-calorie diet and exercise less. On the other hand, the subjects included in this study were a healthy physical examination population who generally have good economic conditions. The high prevalence of MAFLD should be taken seriously. It is important to have an accurate, effective, convenient and low-cost method for screening MAFLD. Therefore, this study explored the correlations between seven obesity measurement indexes and MAFLD and compared their screening accuracy for MAFLD. The seven obesity measurement indexes used in this study included three traditional indexes (BMI, WC, WHtR) and four new indexes (ABSI, BRI, VAI, LAP).

Both ABSI and BRI are new indexes to describe human body shape, which are calculated based on traditional indexes such as WC and BMI. Since body shape seems to be an important risk factor for premature death in the general population, Krakauer first proposed and established ABSI based on BMI and WC, which was initially used to predict the risk of death (19). A study published in 2016 showed that ABSI had a stronger association with total, cardiovascular and cancer mortality (20). In recent years, some researchers have also studied the correlation between ABSI and metabolic diseases. A recent study in 2021 showed that ABSI was significantly associated with cardio-ankle vascular index (CAVI) and the presence of MetS in the middle-aged population and helped to identify individuals with MetS and increased CAVI. ABSI could serve to identify individuals with MetS and increased arterial stiffness (21). After the concept of MAFLD was proposed in 2020, a research team from Sun Yat-sen University in China was the first to study the screening ability of anthropometric indexes for MAFLD, including BMI, WC, WHtR, ABSI, and BRI (22). Their study showed that the AUC of different indexes screening for MAFLD in males were as follows in descending order: BMI (0.81) >WC (0.79) >WHtR (0.77) =BRI (0.77) >ABSI (0.55). The AUCs of the different indexes above for female MAFLD patients was generally consistent with those of male MAFLD patients, but the AUC values were slightly lower than those of male MAFLD patients. Our study also included the above five indexes, and the AUC of each index indicating its screening ability for MAFLD was as follows in descending order: BMI (0.89) >WHtR (0.85) >WC (0.84) >BRI (0.78) >ABSI (0.50). Our study was generally consistent with theirs. Both our study and their study showed that the traditional indexes BMI, WC and WHtR had a certain screening ability for MAFLD, and BMI exhibited the best ability. The new index BRI also had a certain accuracy for screening MAFLD, with an AUC higher than 0.7, but ABSI had poor screening ability for MAFLD. Neither ABSI nor BRI exceeded the ability of the traditional BMI.

The new index ABSI and BRI based on morphologic measurements did not exceed the traditional index for screening MAFLD. Therefore, in addition to the above indexes, our study added two new indexes, LAP and VAI, which take into account both the external morphologic index and internal lipid metabolism-related index in the calculation. Theoretically, LAP and VAI could better reflect the degree of accumulation of body or visceral fat. Several researchers have studied the correlation between VAI and LAP and metabolic diseases, such as MetS, prediabetes and diabetes, metabolic-related cardiovascular disease, and polycystic ovary syndrome (23–28). As mentioned above, ABSI will not be described again due to its poor accuracy for screening MAFLD. Therefore, this section will mainly discuss the correlation between MAFLD and the other six indexes and compare their screening performance for MAFLD. Overall, our study showed that MAFLD had a strong correlation with the traditional index BMI and the new index LAP (r>0.6) and had a moderate correlation with the traditional indexes WC and WHtR and the new indexes VAI and BRI (0.4<r<0.6). ROC curve analysis showed that compared with BMI, the AUC of the other six indexes was statistically significant (*p*<0.001), but there was no statistically significant difference between LAP and BMI in females (p=0.14). ROC curve analysis showed that BMI had the highest AUC for MAFLD (0.84 in males and 0.89 in females), and LAP had a better AUC for MAFLD (0.81 in males and 0.88 in females) than the other indexes. The latest study in 2021 analyzed the correlation between NAFLD and various indexes, including lipid metabolism-related index, LAP, BMI, etc., according to the old diagnostic criteria of NAFLD and found that lipid metabolism-related index and LAP had better screening ability for NAFLD than BMI; the AUCs of LAP and BMI were 0.8659 and 0.8577, respectively (*p*<0.05) (15). In contrast to the study above, our study found that the accuracy of LAP for screening MAFLD did not exceed that of BMI according to the new diagnostic criteria for MAFLD. Stratified by sex, the accuracy of LAP and BMI for screening MALFD in females was similar, but LAP did not exceed BMI. In our study, we found that the accuracy of BMI and LAP for screening MAFLD was higher in females than in males. We believe that this fundamental difference may be attributed to the more complex risk factors for MAFLD present in males. One factor that should not be overlooked is that men typically have a higher alcohol consumption than women. This difference in lifestyle habits may play a crucial role in the observed discrepancy between the sexes regarding the effectiveness of BMI and LAP for MAFLD screening.

It is worth noting that most MAFLD patients have co-existing overweight/obesity status; however, MAFLD is common in populations without obesity, especially in Asia. Due to the different criteria used to define obesity in different countries and regions, according to different literature reports, the prevalence of MAFLD in populations without obesity is as high as 5-45% (29–31). This study showed that the prevalence of MAFLD in population without obesity was 10.87%. At meantime, compared with BMI (AUC 0.76), LAP and VAI had excellent screening accuracy for MAFLD in populations without obesity, LAP had a slightly higher AUC than VAI (0.90 vs. 0.88, *p*< 0.05).

This study showed that among the entire population, traditional BMI had the highest screening accuracy for MAFLD with an AUC of 0.89, which was better than LAP (0.87) and VAI (0.79). In fact, stratified by BMI, LAP (AUC 0.76) and VAI (AUC 0.72) did not demonstrate significantly better screening accuracy for overweight or obese populations (BMI ≥ 23 kg/m^2^) compared to BMI (AUC 0.75). Considering the simplicity and convenience of BMI measurement, which does not require additional laboratory tests, BMI should be preferentially considered for the general population. Strictly speaking, LAP and VAI can certainly be used as well. Additionally, in the non-obese population (BMI< 23 kg/m^2^), both LAP (AUC 0.90) and VAI (AUC 0.88) showed significantly improved screening ability compared to BMI (AUC 0.76). Therefore, in the general population, the first step is to use BMI for screening, as its effectiveness is superior to LAP and VAI. In the overweight or obese population, further screening using LAP or VAI may yield better results. While LAP and VAI had excellent screening accuracy for MAFLD in non-obese populations, LAP had a slightly higher AUC than VAI (0.90 vs. 0.88, *p*<0.05) and required fewer variables for calculation (WC and TG) compared to VAI (WC, TGs, height, weight, and HDL-C), making LAP a more convenient and cost-effective option. Therefore, LAP could serve as an accurate, efficient, convenient, and low-cost screening index for MAFLD in non-obese populations. To sum up, for the general population, the first step is to calculate BMI. Individuals with overweight or obesity (BMI ≥23 kg/m^2^) should be directly classified as high-risk for MAFLD. The second step is to calculate LAP for those without obesity (BMI< 23 kg/m^2^), and those with LAP ≥ 20.75 should also be considered high-risk for MAFLD.

Currently, there is no consensus on universal screening methods for MAFLD, despite the significant health burden it poses. European guidelines support screening for MAFLD in high-risk patients with obesity or metabolic syndrome, while the American Association for the Study of Liver Disease (AASLD) questions the utility of routine screening for MAFLD in these high-risk individuals due to the lack of cost-effective tests and established effective pharmacologic treatments (32). However, new guidelines from the Asian-Pacific Association for the Study of the Liver (APASL) have been developed for the diagnosis and management of MAFLD, as mentioned earlier in our manuscript (5). The guideline base the diagnosis of MAFLD on the detection of fatty liver in conjunction with at least one of three criteria: overweight/obesity, T2DM, or clinical evidence of metabolic dysfunction (including waist circumference, blood pressure, blood lipids, blood glucose, HOMA-IR, hS-CRP, etc.). Fatty liver can be confirmed through various methods, such as ultrasound, transient elastography, regular MRI scanners applying magnetic resonance proton density fat fraction (MR-PDFF), magnetic resonance spectroscopy (MRS), liver biopsy, or serum biomarkers. However, this screening strategy for MAFLD is both time-consuming and costly, making it unsuitable for widespread implementation in the general population.

This study offers a non-invasive, cost-effective, and easily accessible method for screening high-risk populations for MAFLD with good sensitivity and specificity. This approach provides both patients with MAFLD and healthcare professionals with a convenient and persuasive tool for predicting MAFLD risk, and has significant implications for promoting individual stratification management in the realm of health economics. In conclusion, our research contributes to the existing knowledge on obesity measurement indexes and provides guidance for MAFLD screening. By proposing a method that is accurate, efficient, and cost-effective, we aim to support healthcare professionals and patients in identifying high-risk individuals for MAFLD more effectively. We believe our study has the potential to improve both individualized management and overall public health outcomes in the context of MAFLD.

The advantage of this study is that it is the first study to evaluate the correlation between obesity measurement indexes and MAFLD and compare their screening ability for MAFLD by using a healthy physical examination population in northeast China since the concept of MAFLD was proposed. As many as seven indexes, covering traditional and new indexes, were included. In addition, this study defined a population at high risk for MAFLD in a simple way and proposed a new screening strategy for MAFLD. Of course, there are also shortcomings in this study: 1) The design of this study is cross-sectional, which results in some limitations regarding causal inference; 2) The subjects of this study were mostly healthy people who underwent physical examination and could not represent the general population; 3) The determination of the cut-off value of different obesity measurement indexes may be affected by the study subjects and different regions, so it needs to be further verified in the multi-center study; 4) The research center did not carry out HOMA-IR and hs-CRP tests, and some subjects could not be diagnosed as MAFLD according to existing evidence and were excluded; 5) Whether obesity measurement indexes are related to the severity of MAFLD also deserves further investigation. The shortcomings will be further improved in future research.

## 5 Conclusion

**Figure 5:**
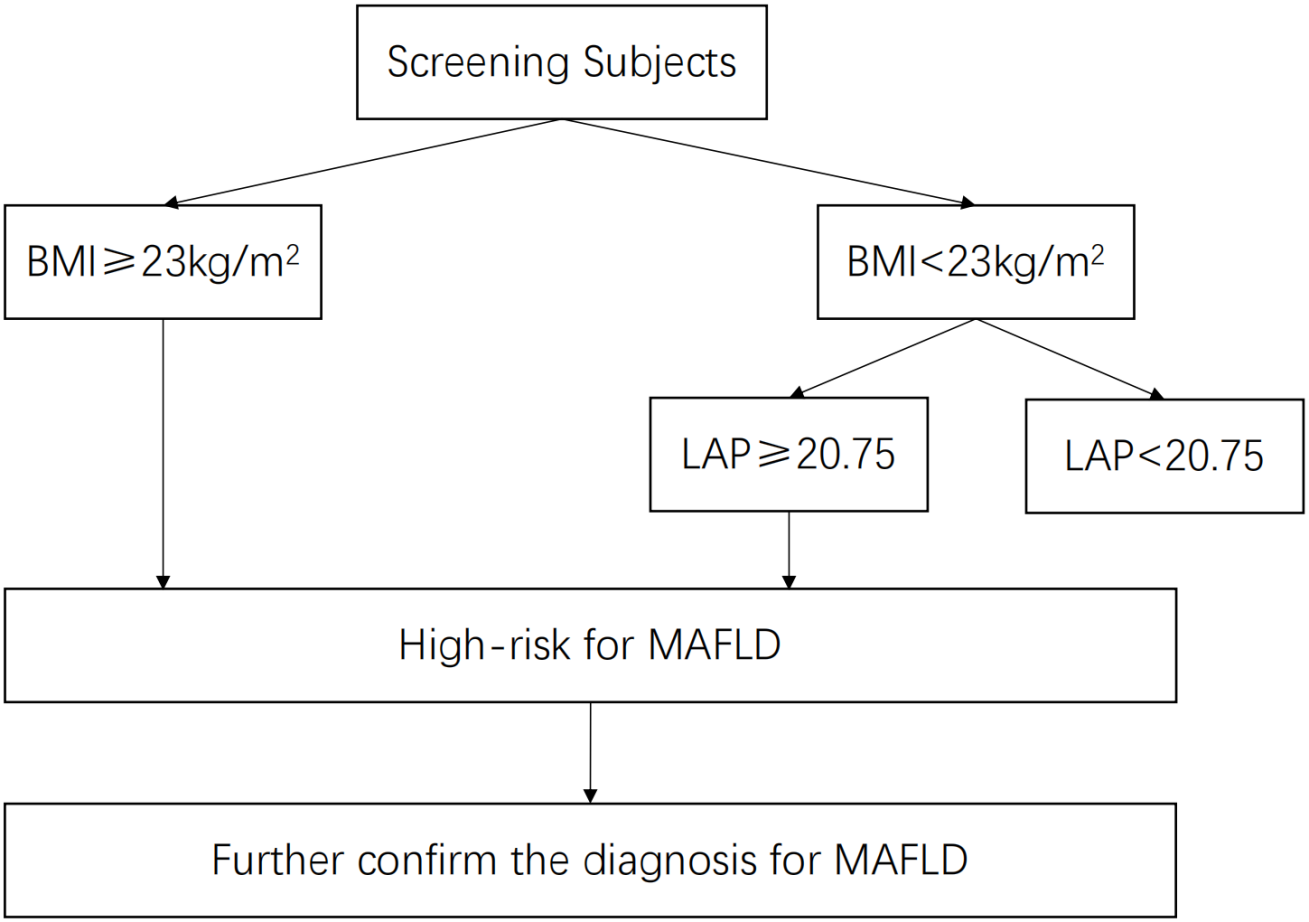
A two-step screening strategy or a high-risk population for MAFLD.

In conclusion, regarding screening for MAFLD in the whole population, the traditional index BMI had the highest accuracy, followed by the new index LAP. However, when screening for MAFLD in populations without obesity (BMI< 23 kg/m^2^), LAP had the highest accuracy, and the optimal cutoff value was 20.75, with a sensitivity and specificity of 85.9% and 79.0%, respectively. Therefore, we proposed a two-step screening strategy for MAFLD, combining BMI and LAP, and defined a high-risk population for MAFLD as follows: 1) BMI ≥ 23 kg/m^2^; and 2) BMI< 23 kg/m^2^ and LAP ≥ 20.75 (as shown in **Figure 5**).

## Data availability statement

The datasets presented in this article are not readily available because of ethical and privacy restrictions. Requests to access the datasets should be directed to the corresponding authors.

## Ethics statement

The studies involving human participants were reviewed and approved by The Ethics Committee of the Second Affiliated Hospital of Harbin Medical University. Written informed consent for participation was not required for this study in accordance with the national legislation and the institutional requirements.

## Author contributions

HW and YZ contributed equally to this work and share first authorship. HW, BQ and CY designed the research. HW, HL, RX and HF acquired the data. HW, YZ and YL analyzed and interpreted the data. HW and YZ drafted the manuscript. HW, YL and BQ made critical revision of the manuscript for important intellectual content. All authors contributed to the article and approved the submitted version.
