# Supplementary figures and images for: Comparison between traditional and new obesity measurement index for screening metabolic associated fatty liver disease
Source: Front Endocrinol (Lausanne). 2023 Apr 21;14:1163682. doi: 10.3389/fendo.2023.1163682 (PMC10160459; doi:10.3389/fendo.2023.1163682)

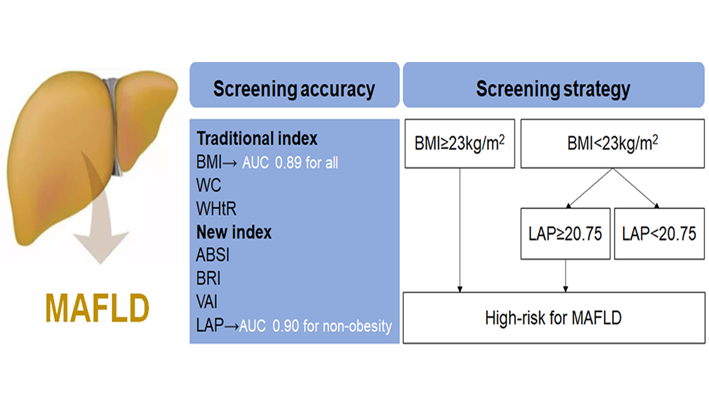

Supplement: Supplementary file 1 [file Image_1.tif]
