# Supplementary material for: Comparison between traditional and new obesity measurement index for screening metabolic associated fatty liver disease
Source: Front Endocrinol (Lausanne). 2023 Apr 21;14:1163682. doi: 10.3389/fendo.2023.1163682 (PMC10160459; doi:10.3389/fendo.2023.1163682)
Supplement: Supplementary file 2 [file DataSheet_1.pdf]

frontiers

Supplementary Material

Comparison between Traditional and New Obesity Measurement Index for Screening Metabolic Associated Fatty Liver Disease

Hongyan Wang†, Yuxue Zhang†, Yupeng Liu, Hui Li, Ruiling Xu, Hongmei Fu, Chaoqi Yan*, Bo Qu*

***Co-corresponding author:**

Bo Qu. Email: qubo_1970@163.com

Chaoqi Yan. Email: yanchaoqi2002@163.com

Supplementary Figures

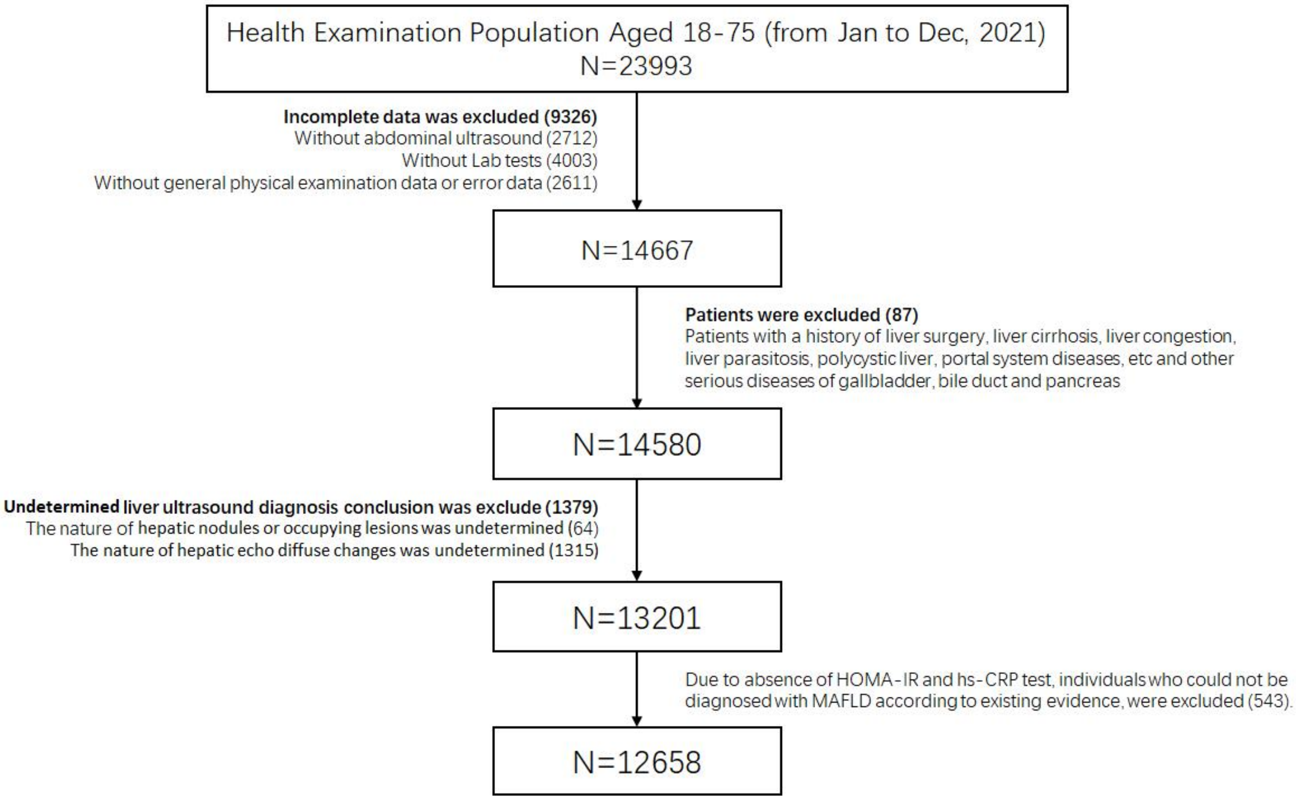

Flow chart

Supplementary Tables

Spearman's rank correlation of MAFLD with seven obesity measurement indexes

Group	statistics	Traditional Indexes			New Indexes			
		BMI	WC	WHtR	ABSI	BRI	VAI	LAP
Total (n=12658)	r	0.67	0.59	0.60	0.01	0.49	0.50	0.64
	p	< 0.001	< 0.001	< 0.001	0.400	< 0.001	< 0.001	< 0.001
Male (n=5952)	r	0.54	0.42	0.43	-0.17	0.36	0.39	0.48
	p	< 0.001	< 0.001	< 0.001	< 0.001	< 0.001	< 0.001	< 0.001
Female (n=6706)	r	0.66	0.59	0.60	-0.02	0.57	0.54	0.65
	p	< 0.001	< 0.001	< 0.001	0.101	< 0.001	< 0.001	< 0.001

BMI: body mass index; WC: waist circumference; WHtR: waist-height-ratio; ABSI: a body shape index; BRI: body roundness index; VAI: visceral adiposity indicators; LAP: lipid accumulation product. r refers to correlation coefficient. n refers to the total number of each group.

Comparison of the AUC values among different obesity measurement indexes for MAFLD

Group	statistics	Traditional Indexes		New Indexes			
		WC	WHtR	ABSI	BRI	VAI	LAP
Total (n=12658)	Z	16.55	14.41	66.16	27.49	21.39	5.82
	p	< 0.001	< 0.001	< 0.001	< 0.001	< 0.001	< 0.001
Male (n=5952)	Z	12.31	11.50	36.98	16.19	11.38	5.36
	p	< 0.001	< 0.001	< 0.001	< 0.001	< 0.001	< 0.001
Female (n=6706)	Z	10.65	8.10	46.64	11.39	11.53	1.46
	p	< 0.001	< 0.001	< 0.001	< 0.001	< 0.001	0.14

BMI was used as a reference standard. AUC: area under the curve. BMI: body mass index; WC: waist circumference; WHtR: waist-height-ratio; ABSI: a body shape index; BRI: body roundness index; VAI: visceral adiposity indicators; LAP: lipid accumulation product; n refers to the total number of each group.

frontiers
